# Joint Detection of Community and Structural Hole Spanner of Networks in Hyperbolic Space

**DOI:** 10.3390/e24070894

**Published:** 2022-06-29

**Authors:** Qi Nie, Hao Jiang, Si-Dong Zhong, Qiang Wang, Juan-Juan Wang, Hao Wang, Li-Hua Wu

**Affiliations:** 1 Electronic Information School, Wuhan University, Wuhan 430072, China; nieqi@whu.edu.cn (Q.N.); qiangwang@whu.edu.cn (Q.W.); 2 School of Business Administration, Zhongnan University of Economics and Law, Wuhan 430073, China; wangjj@zuel.edu.cn; 3 Wuhan Second Ship Design and Research Institute, Wuhan 430064, China; whuwanghao@163.com (H.W.); wlhcheers@126.com (L.-H.W.)

**Keywords:** complex networks, hyperbolic embedding, community detection, structural hole spanner

## Abstract

Community detection and structural hole spanner (the node bridging different communities) identification, revealing the mesoscopic and microscopic structural properties of complex networks, have drawn much attention in recent years. As the determinant of mesoscopic structure, communities and structural hole spanners discover the clustering and hierarchy of networks, which has a key impact on transmission phenomena such as epidemic transmission, information diffusion, etc. However, most existing studies address the two tasks independently, which ignores the structural correlation between mesoscale and microscale and suffers from high computational costs. In this article, we propose an algorithm for simultaneously detecting communities and structural hole spanners via hyperbolic embedding (SDHE). Specifically, we first embed networks into a hyperbolic plane, in which, the angular distribution of the nodes reveals community structures of the embedded network. Then, we analyze the critical gap to detect communities and the angular region where structural hole spanners may exist. Finally, we identify structural hole spanners via two-step connectivity. Experimental results on synthetic networks and real networks demonstrate the effectiveness of our proposed algorithm compared with several state-of-the-art methods.

## 1. Introduction

**Figure 1:**
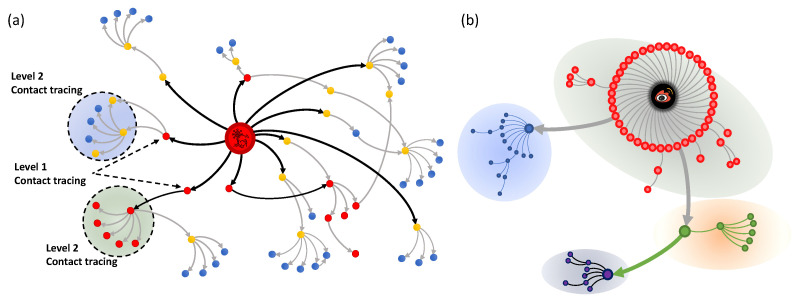
Diffusion networks. (**a**) Epidemic network. Blue nodes represent the susceptible, yellow nodes represent the exposed, and red nodes represent the infected. Directed edges represent the potential transmission path of the contact network. The dotted circle indicates the potential transmission community. (**b**) Information diffusion network. Directed edges indicate the repost (retweet) path of social platforms such as Weibo. Different colored areas represent different communities.

From transportation to information diffusion, networked systems provide an effective way to describe these real-world behaviors [1,2,3,4,5]. A significant part of network analysis, especially social network analysis, is based on network structure [6]. Community structures that can be regarded as clustering with linkage are of commonness in networks [7,8], and the structural hole spanner (SHS), the node bridging different communities, is often accompanied by community structures. Community detection and structural hole spanner identification, the two flourishing topics in network science, help us to understand the structural mechanism of networked systems, such as social networks, information diffusion networks, etc. In the contact tracing and control of epidemics such as COVID-19, community detection and structural hole spanner identification play a very important role collectively. The restraint of contagions generally focuses on individual behaviors, but collective guidance is also very important. Guiding collective behaviors is less implemented since most studies neglect the mesoscopic structure of transmission networks [9]. Finding the community structure and SHS contributes to the understanding of epidemic spreading in the inner community and the inter community, which is helpful in restraining the further spread. In the process of a pandemic, epidemic diseases spread first within the community and then across the community, which is determined by human behavior patterns. Based on human interactions in physical space and cyberspace, the analysis of social networks is conducive to contact tracing. As shown in Figure 1a, with a network structure such as hierarchy and community, we can first detect these susceptible people with direct contact (“level 1” in the epidemic network) and their direct contacts (“level 2” in the epidemic network), which may help us to contain the spread of infection. In the information diffusion network, information such as rumors is usually formed in a local subnetwork and then spread among different communities. As shown in Figure 1b, information transmission consists of inner-community spread and cross-community spread. If the path of cross-community transmission can be found and controlled as soon as possible, further spreading will be effectively contained. In other words, if we can quickly detect the community structure and identify the intermediary nodes between different communities of the diffusion network, we may effectively prevent the further spread of rumors [10].

The above applications show the significant importance of community detection and structural hole spanner identification. From the perspective of network structure, the community is defined from the mesoscopic structure, and SHS is an important node described at the microscale level. Microscopic structural features coexist with mesoscopic structural features in real-world networked systems [11]. To some extent, mesoscopic and microscopic properties of networks are able to determine the dynamics of complex networks collectively [12].

However, most existing research on these two issues has been performed separately, which has ignored the structural correlation between mesoscale and microscale. In topological space, the network modeling approach is intrinsically constrained by the fact that it can only account for pairwise interactions, which makes the structural relation between mesoscale and microscale elusive. As a result, some existing methods suffer from low accuracy and high computational costs. To solve this problem, we use network representation learning (NRL) to embed the network into the geometric space for analysis. NRL is conducted to represent the node or the linkage of a network in low-dimensional spaces [13]. In geometric space, the relationship between nodes or edges of a network can be measured by a certain distance, which may provide metrics to detect communities and structural hole spanners simultaneously.

In recent years, research on hyperbolic spaces has gained much attention in network science [14,15,16,17]. Hyperbolic space is a geometry space of constant negative curvature that can be used to represent the generation of scale-free networks [18]. A common characteristic of many real-world networks is that their degree distributions fit a power-law distribution [19,20,21], which is the premise of embedding networks into hyperbolic space. Hyperbolic embeddings are able to preserve the linkage structure of a scale-free network in a low-dimensional space, especially for hierarchical networks with community structures. On the one hand, in geometric networks, the similarity or distance between nodes can be used for the purpose of measuring community structures. Communities and structural hole spanners are related to the representation of similarity. Hyperbolic embedding can represent the similarity in a very low dimension. Hence, we may address these two tasks on the Poincare disk simultaneously after hyperbolic embedding. On the other hand, hyperbolic embedding makes it possible to represent a complex network through efficient and simple visualization.

In the paper, we propose an efficient algorithm SDHE for simultaneously detecting community structures and structural hole spanners of scale-free networks. Specifically, we use the Poincaré disk model, a model of a two-dimensional hyperbolic plane, to embed high-dimensional networks into low-dimensional hyperbolic space, in which, the angular distribution of nodes reveals their communities. Then, the critical gap, which is conducive to obtaining the angular region of structural hole spanners, is analyzed to detect communities of the network in the hyperbolic plane. Moreover, we study the link relationship between the community and structural hole spanners in hyperbolic space. Finally, we identify structural hole spanners via two-step connectivity. The main contributions of this article are highlighted as follows:

- We analyze mesoscopic and microscopic structural features of scale-free networks and study the inter-community connection probability, which is described as the distance between the mesoscopic communities and the microscopic SHS in hyperbolic space.
- By analyzing community structure and structural hole spanners bridging different communities in hyperbolic space, we find that low-dimensional similarity can be used to measure the community and SHS of networks. We obtain the critical gap for detecting communities and the angular region where structural hole spanners may exist.
- Based on the analysis of the critical gap and angular region, we propose an algorithm SDHE for detecting communities and structural hole spanners simultaneously. Experimental results on synthetic networks and real networks testify the effectiveness and efficiency of our proposed algorithm SDHE.

The rest of the article is organized as follows. Section 2 briefly reviews related work on community detection, SHS identification, and hyperbolic embedding. Section 3 introduces some essential notations and definitions of the issue studied in this paper. Section 4 proposes theoretical analyses and algorithm formation. Section 5 discussed the performance of our proposed algorithm. In Section 6, we analyze the rationale for our algorithm and conclude the paper.

## 2. Related Work

In the section, we review and conclude some valid existing methods for community detection, SHS identification, and hyperbolic embedding. In addition, we briefly discuss features of these existing approaches and the advantage of the joint detection of community and SHS.

### 2.1. Community Detection

Community structure, which describes the mesoscopic structure of the complex network, is an important research content [22]. Research topics related to community structure have always been of high concern by scholars. As a common feature in social networks, community structure has been widely used in various fields [23,24]. How to efficiently find the potential community structure and important nodes in the network has become an important issue of network science research [25]. Since Girvan and Newman proposed the concept of community detection by defining modularity [26,27], abundant community detection algorithms have emerged. For example, spectral clustering [28] detects communities using spectrum analysis. Detection algorithms such as Copra [29] and SLPA [30] are proposed based on a similar idea. Walktrap [31] and Infomap [32] are also typical algorithms based on random walk. Random walk is heuristic, so its calculation result is unstable. In addition, some articles focus on the substructure [33] of networks and analyze the consistency [34,35] and inconsistency [36] of networks. The main idea of these methods is to analyze the different mesoscopic substructures of the network and distinguish them. Moreover, some greedy algorithms, such as CNM [37], can achieve a relatively stable hierarchy of the community. However, for an unknown network, these greedy algorithms are time-consuming and NP-hard.

### 2.2. Shs Identification

The sociological concept of SHS was first proposed by Burt [38]. Some research on social networks has taken advantage of the concept of a structural hole in order to investigate the structure of social networks and the information diffusion of social dynamics [39,40,41]. Ordinarily, only when communities are interconnected can they not form structural holes. Some “bridge” edge [42] linking two or more non-overlapping communities can avoid structural holes. However, the “bridge” edge becomes ambiguous when the network contains overlapping communities. Hence, the application of the “bridge” node (or SHS) is more extensive. To find SHSs in social networks, Lou [39] proposed two effective algorithms: HIS and MaxD. The main idea of HIS is to find more nodes connected by opinion leaders in various groups. MaxD is a structural hole spanner discovery algorithm based on maximum flow. However, the computational complexity of their algorithms is large, and the accuracy depends on the linkage and the number of possible SHSs participating in the final comparison. Rezvani et al. [40] validate that the task of identifying top-k SHSs is of NP-hardness and invent efficient and scalable algorithms for finding top-k SHSs. Due to the NP-hardness of the detection of SHSs, an effective quantitative measurement is particularly important. Xu et al. [41] have provided a method to measure the quality of SHS detection, but this measurement method is not suitable for all networked scenarios. Detecting communities and SHSs are two fundamental and significant tasks in the complex network. He et al. [43] have proposed harmonic modularity to jointly detect communities and SHSs due to the entangled topological nature of these two tasks, which is the first attempt to combine community detection and SHS identification. However, this joint method suffers from high computational costs.

### 2.3. Hyperbolic Embedding

Different from Euclidean geometry, hyperbolic geometry is a geometric space of constant negative curvature. In other words, Euclidean spaces expand polynomially, but hyperbolic spaces expand exponentially, which appears to be inherent in many real scale-free networks [44,45]. In hyperbolic space, large scale-free networks, which are similar to tree-like networks, can be represented in a low-dimensional plane. There are many hyperbolic embedding models, such as the Beltrami–Klein (BK) model [46], the hyperboloid model [47], the Poincaré model [48], etc. The Poincaré disk model is widely used in hyperbolic embedding. There are three main types of embedding methods. The first is the embedding method based on maximum likelihood estimation (e.g., HyperMap [14], efficient embedding [49], and so on). The second is the embedding method based on machine learning (e.g., LaBNE [50], coalescent embedding [16], and so on). The last one is the combination of the two methods (e.g., LaBNE+HM [51,52], Mercator [17], and so on). Their features are shown in Table 1.

Whether in information diffusion or epidemic spreading, community structure and SHS are generally important determinants of percolation processes on complex networks. Community and SHS often coexist in networks. However, most existing studies on the two issues have been conducted separately. In a hyperbolic embedding space, these two tasks can be carried out simultaneously, which substantially improves the efficiency of analyzing the network structure.

## 3. Preliminaries

In this section, some main definitions and vital notations are established to simplify the exposition in other sections. Table 2 itemizes some important notations. These definitions and their main properties will be used to formulate the problem discussed in our paper.

### 3.1. Community

Although the definition of a community is not universally accepted, a general understanding is that communities refer to some dense groups in the network [26]. There is a relatively rigorous definition of a community, which is as follows. *g* is set as a subgraph of a graph *G*. The number of nodes of *g* is set as $\left|V_{g}\right|$ and that of *G* is set as $\left|V\right|$. Then, the intra-subgraph density $\rho_{intra}\left(g\right)$ of the *g* is defined as the ratio between internal edges of *g* and the number of all possible edges of *g*:

(1) $$\rho_{intra}\left(g\right)=\frac{\#\mathrm{internal}\;\mathrm{edges}\;\mathrm{of}\;g}{\left|V_{g}\right|\left(\left|V_{g}\right|-1\right)/2}.$$

The inter-subgraph density $\rho_{inter}\left(g\right)$ is defined as

(2) $$\rho_{inter}\left(g\right)=\frac{\#\mathrm{inter}-\mathrm{subgraph}\;\mathrm{edges}\;\mathrm{of}\;g}{\left|V_{g}\right|\left(\left|V\right|-\left|V_{g}\right|\right)}.$$

When the average link density of *g* is appreciably larger than $\rho_{inter}\left(g\right)$ and much smaller than $\rho_{intra}\left(g\right)$, *g* can be considered as a community.

To a certain extent, nodes in the same clustering usually tend to have common properties. For any node $v_{i}\in C_{p}$, if all of its neighboring nodes are in the community $C_{p}$, $v_{i}$ is an internal node of community $C_{p}$. A clarified community structure helps us to understand and analyze the network structure.

### 3.2. SHS and Weak Tie

**Figure 2:**
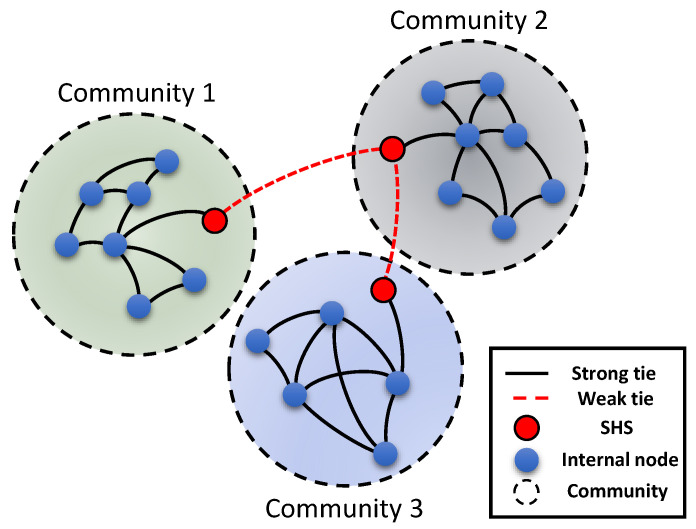
Illustration of weak ties and structural hole spanners.

According to He [43], the SHS has an intuitive definition: for any node $v_{i}\in C_{p}$, if its neighbor $v_{j}\in C_{q}\left(p\neq q\right)$, $v_{i}$ is regarded as an SHS. Shown as the red node in the middle of Figure 2, the structural hole spanner makes different communities bridge through weak ties.

To accurately quantify the SHS, we introduce the definition of the strength of ties. Based on the frequency of interactions, the linkage between two nodes can be divided into two types: strong ties or weak ties. Nodes that have frequent interactions with each other tend to be linked by strong ties. The information flowing through strong ties is usually redundant, which makes it easy for individuals connected by strong ties to form a closed community structure. On the contrary, weak ties can transmit non-redundant information in the process of diffusion. From the perspective of network structure, weak ties are usually the edges connecting different communities, as shown as the dotted line in Figure 2. In some cases, the theory of weak ties proposed by Granovetter [53] is similar to the structural hole theory [38]. They both emphasize the positional relationship of specific nodes in a network. From the perspective of interaction frequency, edges are divided into the strong tie or the weak tie. Strong ties form communities or cliques, whereas weak ties bridge these communities or cliques. If there is a weak tie, structural holes are formed between these different communities. Nodes connected by weak ties are called structural hole spanners. The strength of ties is often used to quantify and judge whether an edge of nodes is a strong tie or a weak tie. In this paper, we use the strength of ties to quantify the edges of a node and then judge whether the node belongs to the structural hole spanners. We introduce the strength of ties in order to compare the experimental results quantitatively [54]. In social networks, strong ties often occur within communities, whereas weak ties occur between different communities. According to the numerical definition of the strength of ties proposed by Zhao et al. [55], the strength of ties $w_{ij}$ is described as:

(3) $$w_{ij}=\frac{c_{ij}}{k_{i}+k_{j}-2-c_{ij}}$$

where $k_{i}$ and $k_{j}$ represent the degrees of $v_{i}$ and $v_{j}$; $c_{ij}$ represents common neighbors of $v_{i}$ and $v_{j}$. If the value of $w_{ij}$ is small, $e_{ij}$ tends to be a weak tie.

## 4. Methods

### 4.1. Hyperbolic Embedding

Hyperbolic embeddings have captured much attention since some scholars have introduced the embedding representation to solve some problems of machine learning [56,57]. The motivation of hyperbolic embeddings is that they can efficiently represent knowledge graphs. The main advantage of hyperbolic embeddings is that graph structures and node attributes can be preserved by very few dimensions.

We used the extended Poincaré disk model to achieve the two-dimensional representation of hyperbolic space. Hyperbolic distances grow exponentially in the hyperbolic space, which is similar to the linkage generation of scale-free networks. Nodes of complex networks are described in the Poincaré disk by the polar coordinate system, i.e., $x_{i}=\left(r_{i},\theta_{i}\right)$, with $r_{i}\in \left[0,+\infty \right)$ and $\theta_{i}\in \left[0,2\pi \right)$ for node $v_{i}$. Based on these polar coordinates, we used geometric distances to represent the similarity between two different nodes. The embeddings of similar nodes should be close, whereas the embeddings of structurally or attributively different nodes should be distant. Popularity and similarity are the main vital features of the embedded networks [58]. Embedded in the Poincaré disk plane, the radial and angular coordinates of nodes represent popularity, and similarity [59], respectively. Hyperbolic embedding can reveal the potential hierarchical structure of scale-free networks. The popularity and the similarity of nodes in hyperbolic space are determined by the existing structure of the network. In other words, once the network structure is given, the radial coordinate and angular coordinate of every node are assigned, respectively. The possibility of a connection between nodes is related to the hyperbolic distance $d_{ij}$, which satisfies

(4) $$\cosh\left(\zeta d_{ij}\right)=\cosh\left(\zeta r_{i}\right)\cosh\left(\zeta r_{j}\right)-\sinh\left(\zeta r_{i}\right)\sinh\left(\zeta r_{j}\right)\cos\left(\Delta \theta_{ij}\right),$$

where $\Delta \theta_{ij}=\pi -\left|\pi -|\theta_{i}-\theta_{j}|\right|$ represents the angular difference of $v_{i}$ and $v_{j}$; $r_{i}$ and $r_{j}$ represent the radial coordinates of $v_{i}$ and $v_{j}$, respectively; $\theta_{i}$ and $\theta_{j}$ represent the angular coordinates of $v_{i}$ and $v_{j}$, respectively; ζ is a constant, and, generally, ζ=1. When $r_{i}$ and $r_{j}$ are large, the hyperbolic distance of $v_{i}$ and $v_{j}$ can be approximated as

(5) $$d_{ij}\approx r_{i}+r_{j}+2\ln\sin\left(\frac{\Delta \theta_{ij}}{2}\right).$$

If the angular coordinates of the two nodes $v_{i}$ and $v_{j}$ are very close (i.e., $\frac{\Delta \theta_{ij}}{2}$ is very small), the above formula can be approximated as

(6) $$d_{ij}\approx r_{i}+r_{j}+2\ln\frac{\Delta \theta_{ij}}{2}.$$

Concretely, we used the efficient embedding (EE) method to embed networks. The EE method can efficiently embed scale-free networks into hyperbolic space, which has achieved a quasi-linear computational complexity [49]. Based on the Poincaré disk model, the main idea of the EE method is to introduce common neighbors in order to obtain the community structure and optimize the node coordinates according to the degree in turn. Specifically, we set G(V,E) as a graph. Embedded by the modified Poincaré disk model, every node has polar coordinates in hyperbolic space. For node $v_{i}$, its radial coordinate $r_{i}$ satisfies

(7) $$r_{i}=\min\left\{R,2\ln\frac{2\left|V\right|\left(\gamma -1\right)T}{k_{i}\sin\left(\pi T\right)\left(\gamma -2\right)}\right\}$$

and

(8) $$R=2\ln\frac{{\left|V\right|}^{2}{\left(\gamma -1\right)}^{2}T}{\left|E\right|\sin\left(\pi T\right){\left(\gamma -2\right)}^{2}},$$

where $\left|V\right|$ represents the total number of nodes; $\left|E\right|$ represents the total number of edges; *R* represents the radius of the extended Poincaré disk; $k_{i}$ represents the degree; γ represents the power-law index; *T* is the temperature coefficient, usually taken as 0.1.

In the EE method, the angular difference between $v_{i}$ and $v_{j}$ is obtained by calculating their common neighbors. The likelihood estimation of angle difference is calculated as

(9) $$\phi \left(c_{ij},r_{i},r_{j}\right)=K\cdot c_{ij}^{\frac{1}{2-\gamma }}\cdot \exp\left(-\frac{1}{2}r_{i}+\frac{r_{j}-R}{2-4\gamma }\right),$$

where $c_{ij}$ represents the number of common neighbors of $v_{i}$ and $v_{j}$; $r_{i}$ and $r_{j}$ represent the radial coordinates of $v_{i}$ and $v_{j}$, respectively; *R* is the radius of the Poincaré disk; *K* is a constant. Embedded in the hyperbolic space by the EE method, the connection possibility $p_{ij}$ of nodes $v_{i}$ and $v_{j}$ satisfies

(10) $$p_{ij}=\frac{1}{1+e^{\frac{\beta \zeta }{2}\left(d_{ij}-R\right)}},$$

where $d_{ij}$ represents hyperbolic distance of $v_{i}$ and $v_{j}$; β and ζ are constant. In this paper, $\beta =\frac{1}{T}=\frac{1}{0.1}=10$ and ζ=1. More details about EE method can be referred to in [49].

### 4.2. Critical Gap of Community Structure

The critical gap in hyperbolic space indicates the angular difference, which is used for partitioning community structures according to angular distribution. When a real network is embedded in a Poincaré disk, the angular distribution of its nodes is not homogeneous. Equation (9) represents that nodes of a network embedded in hyperbolic space have a nature of clustering. The community structure of networks indicates that some densely connected nodes are clustered into corresponding groups. The detection of the community may be a computationally arduous task due to its NP-hardness, but the nature of hyperbolic space contributes to detecting community structures. In hyperbolic space, a pair of nodes with a higher connection probability is more likely to be clustered into the same community. Based on this, a vital characteristic of hyperbolic embeddings is that nodes are considered to belong to the same community when they are distributed in a communal angular area.

Different angular regions are partitioned by a series of critical gaps. In this paper, we used the critical gap method (CGM) [60], a modularity maximization method, to detect potential communities. Modularity [61] is a function measuring the partition quality of community structure. For a particular partition of a network or subnetwork, $s_{i}s_{j}=1$ if nodes $v_{i}$ and $v_{j}$ are in the same community; $s_{i}s_{j}=-1$ if nodes $v_{i}$ and $v_{j}$ are in different communities. Modularity is defined as

(11) $$Q=\frac{1}{4\left|V\right|}\sum_{ij}\left(A_{ij}-\frac{k_{i}k_{j}}{2\left|V\right|}\right)\left(s_{i}s_{j}+1\right),$$

where $\left|V\right|$ represents the total number of nodes; $A_{ij}$ represents the number of edges connecting nodes $v_{i}$ and $v_{j}$; $k_{i}$ and $k_{j}$ represent the degrees of nodes $v_{i}$ and $v_{j}$.

**Figure 3:**
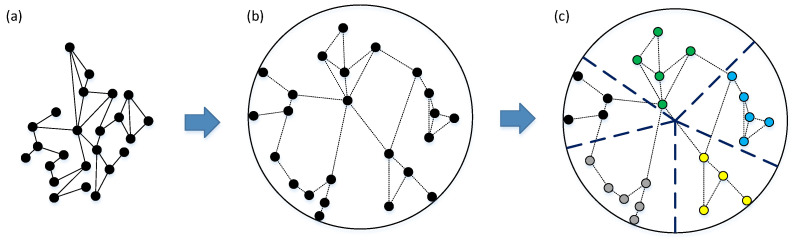
Flow chart of the critical gap method (CGM). (**a**) A network example. (**b**) The network embedded in a Poincaré disk. The dotted line represents the connected shape of Euclidean space, not the actual connection state of hyperbolic space. In fact, the connected edges should be represented as curved lines in hyperbolic space. (**c**) Different communities in hyperbolic space. Different communities are separated by dark blue dashed lines, and the internal nodes in different communities are distinguished by different colors.

Specifically, the CGM detects communities by discovering the best partition of a scale-free network in hyperbolic space [15,62]. The main idea of CGM is to find the appropriate critical gap $\Delta \theta_{c}$ that can separate two consecutive nodes with a little connection possibility. If the angular difference $\Delta \theta_{ij}$ of two consecutive nodes $v_{i}$ and $v_{j}$ is more than the critical gap $\Delta \theta_{c}$, we can believe that the two nodes belong to two different communities, respectively. We gradually increased the value of $\Delta \theta_{c}$ until modularity *Q* no longer became larger. The procedure of CGM is shown in Figure 3, and the pseudo code of CGM is illustrated in Algorithm 1. It shows that a network embedded in a Poincaré disk is divided into different communities.
**Algorithm 1** Critical Gap Method (CGM)**Input:** Graph G=(V,E); Coordinates $\left(r_{i},\theta_{i}\right)$ in hyperbolic plane for $v_{i}\in V$;**Output:** Assignments to communities *C*; Modularity *Q*; 1: **repeat** 2:  Make all pairs of nodes $\left(v_{i},v_{j}\right)$ connected to be a connected component when $\Delta \theta_{ij}\leq \Delta \theta_{c}$; 3:  Assign all nodes of the same connected component to the same community; 4:  Calculating *Q* according to Equation (11); 5:  **if** $Q>\tilde{Q}$ **then** 6:   $\tilde{Q}\leftarrow Q$ 7:  **end if** 8:  Increase $\Delta \theta_{c}$; 9: **until**
$Q<\tilde{Q}$


In fact, the critical gap of a network in hyperbolic space has a theoretical approximation of community detection. Using this theoretical value of the critical gap can not only improve the computational efficiency of CGM but also integrate community detection and SHS identification in hyperbolic space. In different generative models, the value of the critical gap is slightly different [63]. In this paper, we demonstrated the theoretical values of the critical gap under two common models.

(1) In the GPA model

The GPA generative mechanism of networks is considered to give rise to soft communities. In GPA model, the critical gap $\Delta \theta_{c}$ is the expected value of the largest gap. We assumed that the largest gap $\Delta \theta_{\left(n\right)}=max\left\{\Delta \theta_{1},\ldots ,\Delta \theta_{n}\right\}$ where $n=\left|V\right|$ represents the total number of nodes and $\theta_{1},\ldots ,\theta_{n}∼U\left[0,2\pi \right]$ are randomly assigned. If nodes are distributed uniformly, no community structure exists and the model is equivalent to the original popularity-similarity optimization (PSO) model. According to [64], for adequately large value of *n*, $\left\{\theta_{1},\ldots ,\theta_{n}\right\}$ can be approximately regarded as a distribution of the Poisson point process and its density $\lambda =\frac{n}{2\pi }$. Here, the distribution of the angular gaps was approximately exponential with rate λ. Then, the largest gap $\Delta \theta_{\left(n\right)}$ had a probability density function (PDF) such that $f_{\Delta \theta_{\left(n\right)}\left(x\right)}=\frac{n^{2}}{2\pi }e^{-\frac{n}{2\pi }x}{\left(1-e^{-\frac{n}{2\pi }x}\right)}^{n-1}$. Finally, we gained the expected value that

(12) $$\begin{array}{cc} \Delta \theta_{c} & =\frac{n^{2}}{2\pi }\int_{0}^{\infty }xe^{-\frac{n}{2\pi }x}{\left(1-e^{-\frac{n}{2\pi }x}\right)}^{n-1}dx\\ & \approx \frac{2\pi \ln n}{n}. \end{array}$$

(2) In the nPSO model

Nodes in the nPSO model were assumed to satisfy the Gaussian mixture distribution [65]. It is known that the mean value of each community determines the central location of the community and the standard deviation of each component determines the distribution of communities in the angular space. Specifically, a small standard deviation results in isolated communities, and a high value of standard deviation tends to form some overlapped communities. We assume that nodes in hyperbolic space satisfy the Gaussian mixture with equal proportions. Then, their angular coordinates are approximately Gaussian. In that way, their angular differences satisfy the folded normal distribution. Further, the distribution is approximately viewed as an exponential distribution with $\frac{1}{\lambda }=\sigma_{t}\sqrt{\frac{2}{\pi }}\exp\left(-\frac{\mu_{t}^{2}}{2\sigma_{t}^{2}}\right)-\mu_{t}\left[1-2\Phi \left(\frac{\mu_{t}}{\sigma_{t}}\right)\right]$ where $\mu_{t}=\mu ,\sigma_{t}=\sigma \left(t=1,\ldots ,|C|\right)$ if the angular gaps are not too small. Similarly, we have the critical gap, which is as follows:

(13) $$\begin{array}{cc} \Delta \theta_{c} & \approx \frac{\ln n}{\lambda }\\ & =\ln n\cdot \left[\sigma \sqrt{\frac{2}{\pi }}\exp\left(-\frac{\mu^{2}}{2\sigma^{2}}\right)-\mu \left(1-2\Phi (\frac{\mu }{\sigma })\right)\right], \end{array}$$

where μ and σ are constant mean value and standard deviation, respectively.

### 4.3. Angular Area of SHS

Hyperbolic embedding transforms topological analysis of a network into geometric analysis of the network, which is conducive to study network structure by using geometric analysis methods. Based on geometric characteristics of a network, the similarity of two nodes can be measured by geometric distance, such as hyperbolic distance. The polar coordinates of each node in hyperbolic space can provide an index, which can highly improve the efficiency of searching nodes. Having coordinates of each node in hyperbolic space, we can achieve joint detection of community and SHS with low computational complexity.

**Figure 4:**
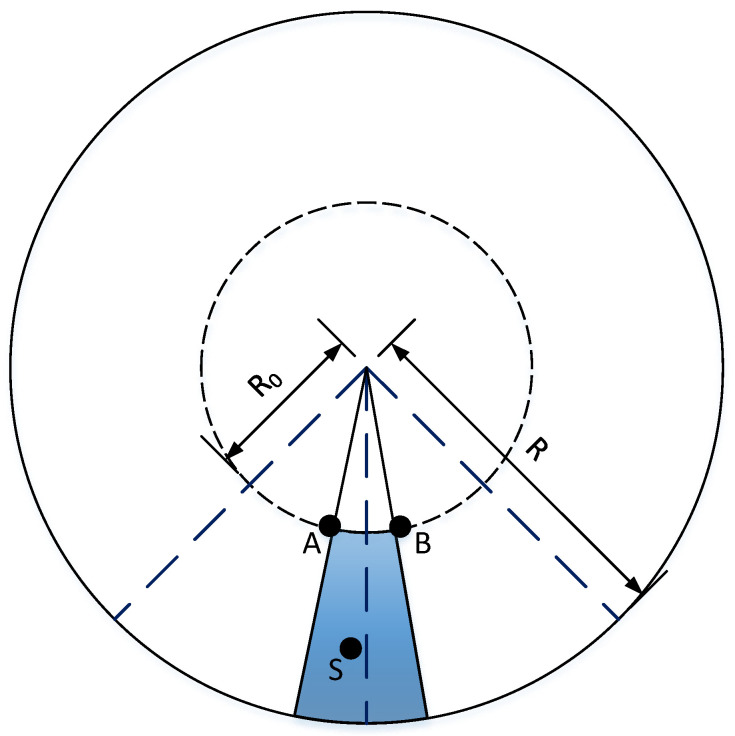
Possible positions of structural hole spanners. Dark blue dashed lines separate the two communities. Node *S* is arbitrarily set as an SHS; then, the shadow area is what we need to calculate.

Specifically, by analyzing the geometry of networks embedded in hyperbolic space, we obtained the critical gap of different communities and the angular region of structural hole spanners bridging the communities. As shown in Figure 4, we assumed that the radius coordinate range of structural hole spanners was from $R_{0}$ to *R*, and the angular coordinate range of SHS was from $\theta_{A}$ to $\theta_{B}$. An arbitrary SHS was set as the node *S*. *A* and *B* are the two nodes closest to *S* outside the region. It is easy to prove that *A* and *B* are on the same geodesic line. The distance between *A* and *B* is

(14) $$d_{AB}\approx R_{0}+R_{0}+2\ln\frac{\Delta \theta_{AB}}{2}=2R_{0}+2\ln\frac{\Delta \theta }{2}.$$

Therefore, the probability of connection between *A* and *B* is

(15) $$p_{AB}=\frac{1}{1+e^{\frac{\beta \zeta }{2}\left(2R_{0}+2\ln\frac{\Delta \theta }{2}-R\right)}}.$$

We assumed that Δ$\theta_{S}$ = $\left\{\Delta \theta_{1},\ldots ,\Delta \theta_{s},\ldots ,\Delta \theta_{\left|C\right|}\right\}$ represents the set of structural hole spanners’ angle range between two nearby communities. Let the maximum value of the probability $p_{AB}$ equal *a*; then, we have the infimum of Δ$\theta_{S}$ as the following:

(16) $$\inf\Delta \theta_{S}=2{\left(\frac{1-a}{a}\right)}^{\frac{1}{\beta \zeta }}e^{\frac{R}{2}-R_{0}},$$

where *a* is the parameter that determines the lower bound of Δ$\theta_{S}$.

When simulating the generation process of scale-free networks, the nPSO model uses Gaussian mixture model to generate network with community structure [65]. In this paper, we assumed that the node distribution in the hyperbolic disk satisfies the Gaussian mixture (GM) distribution $p\left(x|\theta \right)=\sum_{t=1}^{\left|C\right|}\alpha_{t}\varphi \left(x|\theta_{t}\right)$, where $\left|C\right|$ represents the total number of communities and $\alpha_{t}$ determines the shape of the distribution function. For simplicity, we assumed that each sub distribution $\varphi (x|\theta_{t})$ has the same variance. Then, $\alpha_{t}=\frac{1}{\left|C\right|}$, and $\varphi \left(x|\theta_{t}\right)=\varphi \left(\theta_{t}\right)$. We first calculated the position of the angular coordinates in a single Gaussian distribution. Let $\varphi (\theta_{t})=p$; then, we have $\theta_{t}=\mu_{t}\pm \sqrt{2\sigma_{t}^{2}\ln\frac{1}{\sqrt{2\pi }\sigma_{t}p}}$. In this case, we set $\sigma_{t}=\sigma$, where t=1,…,|C|. Then, the angle range of SHS is

(17) $$\Delta \theta_{s}=\left|\theta_{t+1}-\theta_{t}\right|=\frac{2\pi }{\left|C\right|}-2\sigma \sqrt{-2\ln(\sqrt{2\pi }\sigma p)}.$$

For a complex network of scale-freeness, its degree distribution satisfies $P\left(d=k\right)\propto k^{-\gamma }$, where *k* represents the number of degree. We set $P\left(d=k\right)=bk^{-\gamma }$, where *b* is the coefficient related to the network structure. In hyperbolic space, if node *i* satisfies $r_{i}<R$, we can obtain that its degree $k_{i}=\frac{2\left|V\right|\left(\gamma -1\right)T}{\sin(\pi T)(\gamma -2)}e^{-\frac{r_{i}}{2}}=\frac{2\left|E\right|\left(\gamma -2\right)}{\left|V\right|\left(\gamma -1\right)}e^{\frac{R-r_{i}}{2}}$ by calculating Equations (7) and (8). Furthermore, if γ>1, we have the cumulative distribution function (CDF) of degrees as $F_{X}\left(x\right)=P\left(X\leq x\right)=\int_{-\infty }^{x}bk^{-\gamma }dt=\frac{b}{1-\gamma }x^{1-\gamma }$. Then, the CDF of radial coordinates is $F_{Y}\left(y\right)=\frac{b}{1-\gamma }{\left[\frac{2\left|V\right|\left(\gamma -1\right)T}{\sin(\pi T)(\gamma -2)}\right]}^{1-\gamma }e^{\frac{\gamma -1}{2}\cdot y}$. Thus, $P\left(r>r_{i}\right)=1-P\left(r\leq r_{i}\right)=1-F_{Y}\left(r_{i}\right)=1-\frac{b}{1-\gamma }{\left(\frac{2\left|V\right|\left(\gamma -1\right)T}{\sin(\pi T)(\gamma -2)}\right)}^{1-\gamma }e^{\frac{\gamma -1}{2}\cdot r_{i}}$. We assumed that the minimum value of $r_{i}$ is $R_{0}$, and that $P(r>R_{0})=p$. We set that $h=\frac{b}{1-\gamma }{\left(\frac{2\left|V\right|\left(\gamma -1\right)T}{\sin(\pi T)(\gamma -2)}\right)}^{1-\gamma }$; then, the supremum of Δ$\theta_{S}$ is as follows:

(18) $$\sup\Delta \theta_{S}=\frac{2\pi }{\left|C\right|}-2\sigma \sqrt{-2\ln\left[\sqrt{2\pi }\sigma \left(1-h\cdot e^{\frac{\gamma -1}{2}\cdot R_{0}}\right)\right]}.$$

According to Equations (16) and (18), the angular region of SHSs in hyperbolic space was obtained.

### 4.4. SDHE Algorithm

There are many ways to select possible structural hole spanners. This paper used 2-step connectivity to select possible SHSs in the angular ares. The method estimates the number of pairs of a node’s neighbors that are not pairwise linked. The more that the number of edges means, the more significant the possibility of belonging to an SHS. Two-step connectivity is not used directly for detecting SHSs because it is of high computational complexity. The node of large degree results in 2-step connectivity, which is very time-consuming. In large scale-free networks, nodes that have large degrees are usually called hubs. The degree of the SHS with bridging function is not as large as that of the hubs. When we embedded a large network into hyperbolic space, the hierarchical relationship between nodes was revealed. Specifically, the hubs of larger degrees and structural hole spanners were assigned to different levels in the hyperbolic space. If we exclude the area where the structural hole spanner is impossible to exist, we can avoid wasting much time on computing irrelevant nodes. We used the geometric relations of nodes in hyperbolic space to divide the geometric boundary of structural hole spanners. Then, 2-step connectivity was used to filter needed nodes in the region. When the value of a node is positive, the node is output as a candidate SHS. Finally, the top-k nodes were selected as SHSs. The pseudo code of SDHE is illustrated in Algorithm 2.
**Algorithm 2** SDHE**Input:** Graph G=(V,E); Coordinates $\left(r_{i},\theta_{i}\right)$ in hyperbolic plane for $v_{i}\in V$; The critical gap $\Delta \theta_{c}$;**Output:** The distributed structural hole spanners;  1: Initialize and sort the set of angular coordinates $\left\{\theta_{i}\right\}$;  2: Calculate the consecutive angular differences Δθ = $\left\{\Delta \theta_{ij}\right\}$;  3: Detecting communities by using Algorithm 1 CGM;  4: **for**
$\Delta \theta_{ij}\in \Delta$
θ
**do**  5:     **if** $\Delta \theta_{ij}>\Delta \theta_{c}$ **then**  6:         Select nodes whose angular coordinates satisfy $\theta_{q}\in \left(\theta_{i}-\sup\Delta \theta_{S},\theta_{i}+\sup\Delta \theta_{S}\right)$ or $\theta_{q}\in \left(\theta_{j}-\sup\Delta \theta_{S},\theta_{j}+\sup\Delta \theta_{S}\right)$;  7:         Select top-k nodes with positive 2-step connectivity scores;  8:     **end if**  9: **end for**10: **return** Top-k structural hole spanners;

## 5. Results

In this section, we evaluate the effectiveness of our proposed algorithm in this article. Firstly, we briefly illustrate the network datasets and several compared methods. Then, we present the quality measurement and evaluate the performance of the proposed algorithm on community detection and SHS identification. Finally, we discuss the results.

### 5.1. Datasets

We used three synthetic networks and nine real-world networks to experiment. We utilized the nPSO model [65] to generate synthetic networks. The nPSO model was used for generating some specific networks in hyperbolic space, where heterogeneous angular attractiveness of nodes was preset by means of sampling the angular coordinates from a mixture of Gaussian distributions. We used the nPSO model to generate three networks of different parameters. In addition, nine real-world networks [66] were used in the experiment. Some indicators of these experimental networks are shown in Table 3.

### 5.2. Compared Methods

Based on the CGM, we compared SDHE with the following methods for detecting top-k structural hole spanners.

- PageRank-based (PR) [67] is a classic ranking algorithm that assigns each node a PageRank score for ranking potential SHSs. This algorithm is widely used in industries such as Google Search.
- Betweenness-centrality-based (BC) [68] gives each node a score for its shortest paths. Then, the algorithm selects nodes of the top-k scores as the possible SHSs.
- Two-step connectivity (2-Step) [69] estimates the number of pairs of a node’s neighbors that are not pairwise linked. The more that the number of edges means, the larger the possibility of belonging to an SHS.
- HAM [43] formulates a harmonic modularity function for discovering the possible SHSs. The rationale is that nodes whose neighbors belong to more different subnetworks can be regarded as SHSs.
- ESH [70] is an algorithm that simulates a factor diffusion process in SHS identification. To some extent, the motivation of ESH is similar to that of the label propagation algorithm (LPA) [71].

The complexity of the aforementioned methods is briefly discussed as follows. The complexity of efficient embedding (EE) used for achieving the hyperbolic embedding is $O(\left|V\right|\cdot polylog\left(\left|V\right|\right))$. The computational complexity of modularity *Q* is $O({\left|V\right|}^{2})$, so the CGM runs in $O(\tau \cdot {\left|V\right|}^{2})$, where τ represents the iteration times. For classic community detection algorithms, they usually have a high computational complexity. The Louvain algorithm runs in $O(\left|V\right|\cdot \log^{2}\left|V\right|)$. The complexity of KL is $O(k\cdot {\left|V\right|}^{2}\log\left|V\right|)$. The CNM algorithm runs in $O(\left|V\right|\cdot \log^{2}\left|V\right|)$. After obtaining the hyperbolic coordinates of the network nodes, we can use their geometric relationship to limit the angular area of structural hole spanners to a small angular region, which can greatly improve the efficiency of SHS detection. The computational complexity of the top-k SHS detection algorithm in this paper is $O(\frac{{\left|E\right|}^{\frac{3}{2}}\cdot \log\left|V\right|}{\left|V\right|})$. For some existing SHS detection algorithms, the complexity is often high because of the inevitable global search. For example, the computational complexity of betweenness is $O(\left|V\right|\left|E\right|+{\left|V\right|}^{2}\cdot \log\left|V\right|)$. That of two-step connectivity is $O({\left|E\right|}^{\frac{3}{2}})$. The HAM algorithm runs in $O({\left|V\right|}^{3})$. ESH and PageRank, although they both have a low complexity of $O(k\cdot \left|V\right|)$, are unstable.

### 5.3. Evaluation Criteria

To evaluate the effectiveness of algorithms, two elements should be taken into consideration: the quality of community detection and the accuracy of SHS identification. Commonly, modularity, which is described in Equation (11), is applied to estimate the quality of detecting community structures. In addition, the strength of ties, which is the quantitative measurement of edges, can be used for measuring the accuracy of SHS identification. Strong ties have greater values of strength, whereas weak ties have smaller values. From the point of view of topology, nodes and edges of a graph are equivalent. When a vertex has abundant edges connecting with vertices in one community, it can be considered that the vertex is strongly tied with the community. Specifically, we used the average connection strength of node *i* to quantify the degree of weak ties as follows

(19) $$w_{i}=\frac{1}{C_{nb}\left(i\right)}\sum_{j}w_{ij}$$

where $w_{ij}$ represents the connection strength of $v_{i}$ and $v_{j}$; $C_{nb}\left(i\right)$ represents the number of varied communities that $v_{i}$’s neighbors belong to. The average connection strength reflects connection properties for a specific node. When $w_{i}$ is very small, node $v_{i}$ is more likely to be an SHS.

In this paper, we combine these two evaluation indicators for measuring the effectiveness of joint detection. Similar to the GR-score [16], a CS-score that covers the two tasks has been introduced for the purpose of comparing the performance of methods. It is set as follows.

(20) $$CS_{score}=\frac{Q}{\frac{1}{k}\sum_{i=1}^{k}w_{i}}$$

where *k* represents the number of selected top-k SHSs, $w_{i}$ represents the average connection strength of node *i*, and *Q* represents the modularity. When the CS-score is higher, the performance of community and SHS detection is more effective.

### 5.4. Experimental Result

Some experimental results of our proposed algorithm are discussed in detail in this section.

**Figure 5:**
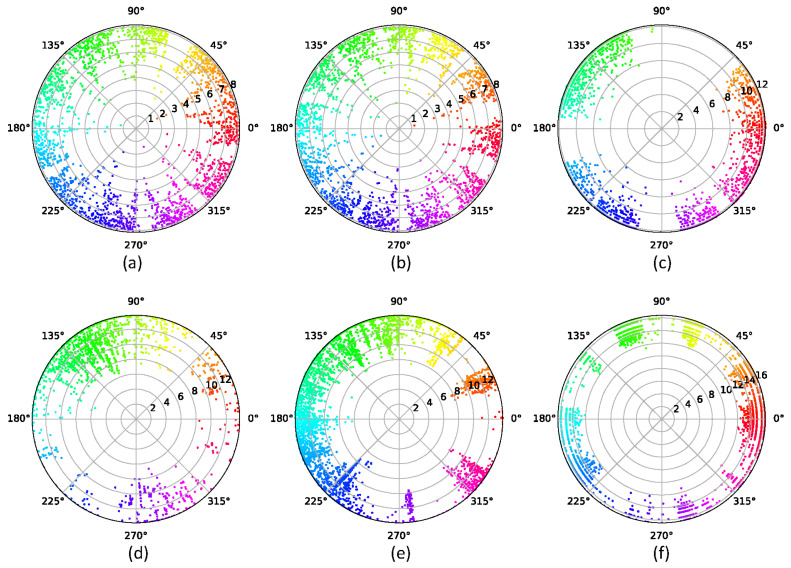
Visualization of network communities in the hyperbolic space. Every point represents a node of a connected graph. The disk takes polar coordinates as the coordinate system. For the sake of simplicity, edges have not been added in the disk. We used the CGM to determine the community structure of the network. The colors are randomly generated, and different colors represent different angular coordinates. (**a**) Synthetic network 1 with Q=0.6520, $\left|C\right|=15$. (**b**) Synthetic network 2 with Q=0.6466, $\left|C\right|=16$. (**c**) Synthetic network 3 with Q=0.8355, $\left|C\right|=20$. (**d**) soc-hamsterster with Q=0.3342, $\left|C\right|=36$. (**e**) fb-pages-politician with Q=0.2929, $\left|C\right|=10$. (**f**) power-US-Grid with Q=0.7576, $\left|C\right|=15$.

We used the EE method to embed networks into hyperbolic space. Figure 5 shows the visualization result of hyperbolic embedding. In hyperbolic space, every point represents a node. The hyperbolic distance of two nodes represents the linkage possibility of the two nodes, and the angular difference between the two nodes indicates the similarity between the two nodes. We used the CGM to detect communities. Nodes in different communities are distinguished by different colors, which represent angular areas. Moreover, we obtained the angular region according to Equation (18) and located it at the community interval divided by CGM. We then used two-step connectivity to select structural hole spanners. Three synthetic networks and nine real network datasets were used to conduct experiments, and the results are listed in Table 4. We compared our method with PageRank [67], betweenness [68], two-step connectivity [69], HAM [43], and ESH [70] for detecting top-k SHSs. Theoretically, SHS is more likely to have a low strength of ties.

It can be seen from Table 4 that our algorithm outperforms other algorithms in all datasets, especially for synthetic networks. Due to ambiguous and overlapping community structures of the synthetic network in the hyperbolic plane, SHS identification by our algorithm can be efficiently achieved. Although the structure of real networks is not as ambiguous as that of synthetic networks, our algorithm applied in real networks has a good effect.

**Figure 6:**
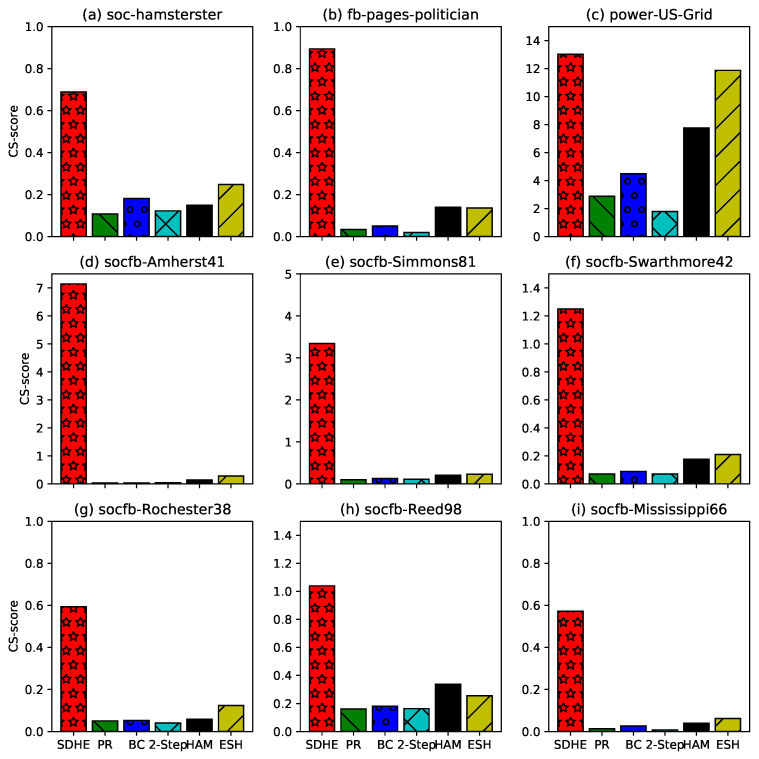
CS-score of different methods on different networks.

To jointly compare the comprehensive performance of community detection and SHS identification, the CS-score is devised to evaluate different algorithms. The formation of the CS-score consists of two parts: modularity and the strength of ties. The results are shown in Figure 6. Based on CGM, the CS-score of our algorithm is higher than that of other algorithms. This indicates that the proposed algorithm in this paper is effective in the joint detection of community and SHS.

## 6. Conclusions and Discussion

In this article, we propose a novel algorithm SDHE for simultaneously detecting communities and structural hole spanners of networks in hyperbolic space. Different from common algorithms, our proposed algorithm can avoid global searches on a large scale. Specifically, with the help of the extended Poincaré disk model to embed nodes of scale-free networks into hyperbolic space, we are able to utilize the critical gap and the angular region to detect communities and SHSs. The CS-score containing the modularity and strength of ties is introduced to evaluate the performance of our proposed algorithm in this paper, and the computational result indicates that our algorithm outperforms other detection algorithms. The main reason is that the network structure is well represented in hyperbolic space. Communities of the network are placed in sectors with different angular ranges in the hyperbolic plane, so it is efficient in detecting communities and structural hole spanners by means of hyperbolic geometry. In other words, our proposed algorithm avoids analyzing nodes near the center of each community, which reduces the computational complexity.

We used the proposed algorithm to analyze synthetic networks and real networks, respectively. The experimental results have shown a great performance in synthetic networks because the generative mechanism of these synthetic networks is consistent with our method. Our method also has a good performance in some real networks, but the precondition is that these real networks are of good community structure in hyperbolic space and are not too sparse. The results indicate that the joint detection of mesoscopic and microscopic structure is effective and efficient in hyperbolic space because hyperbolic embeddings shed light on the hierarchy, community, and linkage of complex networks in a simple low-dimensional plane. In hyperbolic space, the similarity of nodes can be represented by the hyperbolic distance, which provides a metric to analyze the network structure efficiently. Although we spent some time embedding the network into the hyperbolic representation space, it is very necessary and instructive for network analysis.

Overall, the proposed algorithm of detecting communities and structural hole spanners simultaneously in hyperbolic space is effective and efficient, and has good application prospects in the fields of contact tracing, rumor control, and so on. The main drawback of our proposed algorithm is that the total computational complexity is greatly affected by the hyperbolic embedding algorithm. Hence, future possible research achievements could be directed towards developing highly efficient embedding algorithms that can represent real networks quickly and accurately in representation space.

## Figures and Tables

**Table 1:** Comparison of some embedding methods.

Method	Feature	Complexity
HyperMap	Based on PSO model	$O({\left|V\right|}^{3})$
Efficient Embedding	Introducing common neighbors	$O(\left|V\right|\cdot polylog\left(\left|V\right|\right))$
LaBNE	Based on Laplace spectral decomposition	$O({\left|V\right|}^{2})$
Coalescent Embedding	Based on repulsion–attraction and betweenness	$O({\left|V\right|}^{2})$ to $O(\left|E\right|\left|V\right|)$
LaBNE+HM	Combining LaBNE with HyperMap	$O({\left|V\right|}^{2})$ to $O({\left|V\right|}^{3})$
Mercator	$S^{1}$ model	$O({\left|V\right|}^{2})$

**Table 2:** Comparison of some embedding methods.

Notation	Definition
$V={\left\{v_{i}\right\}}_{i=1}^{n}$	the set of nodes; $v_{i}$ represents the *i*-th node
E⊇V×V	the set of edges; $e_{ij}=\left(v_{i},v_{j}\right)$ represents a link
$C={\left\{C_{i}\right\}}_{i=1}^{p}$	the set of communities whose element $C_{i}$ represents community *i*
G(V,E)	an undirected graph (network) that consists of set of nodes *V* and set of edges *E*
$\left|V\right|$ (or *n*)	the total number of nodes
$\left|E\right|$	the total number of edges
$\left|C\right|$	the total number of communities
$r_{i}$	the radial coordinate of node $v_{i}$ in hyperbolic space
$\theta_{i}$	the angular coordinate of node $v_{i}$ in hyperbolic space
$\Delta \theta_{ij}$	the angular difference of $v_{i}$ and $v_{j}$
$d_{ij}$	the hyperbolic distance of $v_{i}$ and $v_{j}$
$\Delta \theta_{c}$	the critical gap, which is an angular gap partitioning two communities
Δ $\theta_{S}$	the set of structural hole spanners’ angle difference range
inf	infimum
sup	supremum
$C_{nb}\left(i\right)$	the number of varied communities that node $v_{i}$’s neighbors belong to
$k_{i}$	degree of $v_{i}$
$w_{ij}$	strength weight of edge connecting $v_{i}$ and $v_{j}$

**Table 3:** Network datasets.

Graph Name	$\left|V\right|$	$\left|E\right|$	Average Degree3	Average Clustering Coefficient	Power-Law Index	Category
Synthetic network 1	2000	98,725	99	0.6016	2.9039	Synthetic networks
Synthetic network 2	2000	98,725	99	0.6157	2.8989	Synthetic networks
Synthetic network 3	2000	7990	8	0.4640	3.4288	Synthetic networks
soc-hamsterster	2000	16,631	14	0.5375	4.8520	Social networks
fb-pages-politician	5908	41,729	14	0.3851	3.2455	Social networks
power-US-Grid	4941	6594	3	0.0801	3.2175	Power networks
socfb-Amherst41	2235	90,954	81	0.3104	5.6425	Social networks
socfb-Simmons81	1510	32,988	43	0.3149	4.7393	Social networks
socfb-Swarthmore42	1657	61,050	73	0.2965	5.5988	Social networks
socfb-Rochester38	4561	161,404	70	0.2932	5.3782	Social networks
socfb-Reed98	962	18,812	39	0.3184	4.3817	Social networks
socfb-Mississippi66	10,519	610,911	116	0.2479	5.4252	Social networks

**Table 4:** Strength of top-k possible SH spanners.

Graph Name	Our Algorithm	PageRank	Betweenness	2-Step	HAM	ESH
Synthetic network 1	**2.2402**	5.5835	5.5835	5.5835	5.5076	3.9269
Synthetic network 2	**4.9280**	10.4967	10.4967	10.4967	10.0240	14.7173
Synthetic network 3	**42.1019**	67.4177	55.5883	60.3040	71.0014	49.4057
soc-hamsterster	**17.4755**	112.2295	66.2897	98.3370	80.4742	48.5466
fb-pages-politician	**33.7559**	901.6482	599.9436	1583.0686	216.4362	221.5029
power-US-Grid	**5.6422**	25.5685	16.3807	41.0428	9.4812	6.1929
socfb-Amherst41	**0.1590**	33.8065	31.1441	28.5272	8.0049	3.9821
socfb-Simmons81	**0.4939**	16.2367	12.9662	15.0613	7.9427	7.1944
socfb-Swarthmore42	**0.5542**	9.7298	7.8113	9.7298	3.9401	3.2916
socfb-Rochester38	**8.9028**	103.4225	99.0418	128.1894	90.4591	42.3957
socfb-Reed98	**1.6438**	10.5912	9.4288	10.4065	5.0676	6.6712
socfb-Mississippi66	**3.1859**	135.9779	67.7351	227.7486	45.1414	29.2449

## Data Availability

Not applicable.

## Abbreviations

The following abbreviations are used in this manuscript:

SHS	Structural hole spanner
NRL	Network representation learning
EE	Efficient embedding
CGM	Critical gap method
GPA	Geometric preferential attachment
PSO	Popularity-similarity optimization
GM	Gaussian mixture
CDF	Cumulative distribution function
